# Impact of Thermal Treatment on Growth Factor Levels in Injectable Horizontal Platelet‐Rich Fibrin: An In Vitro Study

**DOI:** 10.1002/cre2.70353

**Published:** 2026-04-08

**Authors:** Deshetti Ketan, Anita Kulloli, Sharath Shetty, Santosh Martande, Shivani Yerte, Avinash Sanap, Shubham Lawate, Chaitaly Munot, Dileep Sharma

**Affiliations:** ^1^ Department of Periodontology Dr. D.Y. Patil Dental College and Hospital, Dr. D.Y. Patil Vidyapeeth Pimpri, Pune Maharashtra India; ^2^ Department of Regenerative Medicine Laboratory Dr. D.Y. Patil Dental College and Hospital, Dr. D.Y. Patil Vidyapeeth Pimpri, Pune Maharashtra India; ^3^ Oral Health, School of Health Sciences College of Health, Medicine and Wellbeing, University of Newcastle Ourimbah NSW Australia; ^4^ College of Medicine and Dentistry James Cook University Smithfield QLD Australia

**Keywords:** angiogenesis, angiopoietin‐2, growth factors, H‐PRF, injectable PRF, PDGF, platelet‐rich fibrin, regenerative periodontology, thermal treatment, VEGF

## Abstract

**Background:**

Platelet‐rich fibrin (PRF) is widely recognized for its regenerative properties in periodontal therapy, particularly through the release of endogenous growth factors. Injectable horizontal PRF (H‐PRF), a recent advancement, has gained interest due to its improved cell distribution and structural integrity. The aim of this study was to analyze and compare the levels of growth factors released from injectable H‐PRF subjected to different thermal treatments.

**Methods:**

Venous blood samples from 16 healthy participants (aged 18–25 years) were collected and divided into four groups: Group I (unheated control), Group II (37°C), Group III (45°C), and Group IV (60°C). Liquid H‐PRF was prepared using horizontal centrifugation (700 RCF, 8 min), followed by controlled heating. Growth factor quantification was performed using the LEGENDplex Human Growth Factor Panel. Solidification and degradation profiles were also recorded. Statistical analyses included ANOVA and Bonferroni post hoc tests (significance at *p* < 0.05).

**Results:**

The solidification time decreased significantly with increasing temperature, ranging from 24 min in Group I to 5 min in Group IV. After 24 days, heated H‐PRF gels (especially 45°C and 60°C) retained greater mass, indicating slower degradation. Significant intergroup differences were observed for angiopoietin‐2 (*p* < 0.001), PDGF‐AA (*p* = 0.002), TGF‐α (*p* = 0.036), and VEGF (*p* = 0.011), with Group II showing the highest levels of angiogenic factors. No significant differences were noted for EGF, FGF‐basic, HGF, and PDGF‐BB.

**Conclusion:**

Thermal treatment of H‐PRF at moderate temperatures (particularly 37°C and 45°C) enhances the release of angiogenic growth factors while improving the mechanical stability of the gel. However, the heating protocol will need to be customized based on the intended clinical applications.

## 1 Introduction

The recent advancements in periodontal regenerative strategies have been significantly influenced by the integration of biologically active biomaterials and autologous growth factors. Among these, autologous platelet concentrates such as platelet‐rich plasma (PRP) and platelet‐rich fibrin (PRF) have gained considerable attention for their capacity to facilitate soft and hard tissue regeneration through the targeted release of bioactive molecules (Ding et al. 2021). Their relatively simple protocols, cost‐effectiveness, and, above all, their autologous nature have positioned them as a favorable approach in clinical regenerative protocols.

PRP represents the first generation of platelet concentrates, with its regenerative benefits often challenged by the need for anticoagulants that can interfere with the natural coagulation process compromising the biological environment at the application site (Choukroun et al. 2006). To address this limitation, Choukroun et al. introduced PRF as a second‐generation platelet concentrate that excludes anticoagulants during preparation (Choukroun et al. 2006). PRF is produced through a single centrifugation step, resulting in the formation of a fibrin clot enriched with platelets and leukocytes. This matrix functions as a natural scaffold that sustains the gradual release of essential growth factors and supports cellular migration and proliferation during the healing process (Choukroun et al. 2006).

The PRF matrix is rich in several crucial growth factors, including platelet‐derived growth factor (PDGF), vascular endothelial growth factor (VEGF), epidermal growth factor (EGF), transforming growth factor‐beta (TGF‐β), and insulin‐like growth factor‐I (IGF‐I) (Chatterjee and Debnath 2019; Masuki et al. 2016). These signaling molecules exert pleiotropic effects, playing a pivotal role in modulating angiogenesis, matrix remodeling, and differentiation of stem cells in periodontal ligament space, thereby facilitating regeneration of alveolar bone, periodontal ligament, and cementum.

The regenerative efficacy of PRF is closely associated with its physical and biochemical properties, including fibrin architecture, mechanical strength, biodegradation rate, and the kinetics of growth factor release (Ghanaati et al. 2014). Variations in the centrifugation protocol, particularly rotational speed and angle, can significantly influence the composition and functional potential of the PRF obtained. To optimize these parameters, several advanced formulations of PRF have emerged, such as Advanced PRF (A‐PRF), A‐PRF+ , Titanium PRF (T‐PRF), and Injectable PRF (i‐PRF), each tailored to enhance specific regenerative outcomes (Shirbhate and Bajaj 2022).

i‐PRF, a low‐speed, short‐duration centrifuged product, is highly rated for its injectable form and ability to remain in a fluid state for a limited period before clotting (Kawase 2015). These properties allow for efficient integration with graft materials and membranes when used concurrently (Kawase 2015). However, its significant drawbacks, such as rapid degradation rate and limited mechanical resistance, have often led to reduced functional stability at the surgical site.

To address these concerns, horizontal centrifugation has been introduced, resulting in horizontal PRF (H‐PRF). This method utilizes a centrifuge with swing‐out rotors that allow for more effective separation of blood components based on density, resulting in a fibrin matrix with superior cell distribution, enhanced porosity, and increased regenerative potential (Miron et al. 2019). The uniform architecture of H‐PRF is also known to support the sustained release of growth factors, thereby promoting prolonged biological activity (Farshidfar et al. 2025).

Recent explorations in the field have proposed thermal modulation of the plasma fraction post‐centrifugation as a means of enhancing the mechanical integrity and degradation resistance of PRF. Controlled heating at specific temperatures, typically 37°C, 45°C, and 60°C, for 10 min has been shown to induce conformational changes in fibrin proteins, leading to improved gel formation when subsequently mixed with the cellular fraction of liquid PRF (Shirakata et al. 2021; Zheng et al. 2022; Fujioka‐Kobayashi et al. 2020). This composite can potentially yield a structurally stable, bioactive filler suitable for regenerative applications.

Despite its potential benefits, investigations into the effect of thermal treatment on the physicochemical and biological characteristics of injectable H‐PRF remain limited. In particular, the influence of heating on growth factor release profiles, gelation kinetics, and degradation behavior is not yet fully understood. Addressing this gap is essential for optimizing the clinical performance of thermally modified PRF constructs. Hence, the present study aims to analyze and compare the levels of growth factors of injectable H‐PRF at different heating temperatures.

## 2 Methodology

This in vitro study was conducted at the Department of Periodontology and the Regenerative Medicine Laboratory (RML) at Dr. D.Y. Patil Dental College and Hospital, India. The study protocol was approved by the Dr. D.Y. Patil Vidyapeeth Ethics Committee (Approval number‐DPU/806/2/2023), ensuring that all procedures followed the appropriate ethical standards.

A total of 16 blood samples (10 mL each) were collected from healthy adult participants (male and female) aged between 18 and 25 years. Each blood sample obtained from the participants was processed and aliquoted into four experimental groups: control (unheated), 37°C, 45°C, and 60°C. Group I served as the control group, consisting of injectable H‐PRF without heating; Group II included injectable H‐PRF heated to 37°C; Group III included injectable H‐PRF heated to 45°C; and Group IV consisted of injectable H‐PRF heated to 60°C (all for 10 min each).

### 2.1 Patient Selection

Healthy adults aged between 18 and 25 years of both genders with adequate platelet counts and who voluntarily agreed to provide informed consent for both the blood sample collection and participation in the study were included. Exclusion criteria were applied to ensure that participants had no known history of systemic diseases, tobacco use in any form, or abnormal complete blood count. Additionally, participants who were using medications that affect white blood cells or platelets, or blood thinners, were excluded from the study. Prior to blood sample collection, each consenting participant underwent a complete blood count, detailed medical history review, and clinical oral examination. Written informed consent was obtained from all subjects, and blood reports were reviewed to ensure that the participants met the inclusion criteria.

### 2.2 Materials and Instruments

A range of materials and instruments were utilized during the study. Venous blood was collected from the antecubital vein of each participant using 10 mL sterile additive‐free plastic PRF tubes (BD Vacutainer, Becton Dickinson, Franklin Lakes, NJ, USA). The tubes were plain tubes without anticoagulants or silica clot activators to ensure natural coagulation during centrifugation. Blood was drawn directly from the antecubital vein into the centrifugation tubes, and no transfer between tubes was performed to avoid artificial clot activation or delays. Additionally, the time interval between venipuncture and centrifugation was maintained within 60 s to preserve the integrity of the PRF clot formation process.

For the preparation of injectable H‐PRF, venous blood collected in additive‐free PRF tubes was immediately subjected to centrifugation using a horizontal centrifuge (REMI R8C, REMI Elektrotechnik Ltd., Mumbai, India) equipped with a swing‐out rotor system. The samples were centrifuged at 700 × *g* for 8 min, corresponding to approximately 2500 rpm with a rotor radius of 10 cm. The relative centrifugal force was calculated using the formula RCF = 1.118 × 10^−5^ × *r* × (rpm)^2^, where r is the rotor radius in centimeters. Centrifugation was carried out at room temperature (22°C–24°C) using standard acceleration settings and passive deceleration without braking to prevent disruption of the forming fibrin matrix.

Other necessary equipment included insulin syringes, incubators, Eppendorf tubes, tissue‐embedding molds, DMEM (Dulbecco's Modified Eagle Medium), and a 5% CO_2_ incubator for cell culture. For the growth factor analysis, the LEGENDplex Human Growth Factor Panel V02 was used, which enabled the quantification of various growth factors through a multiplex assay using fluorescence‐encoded beads.

### 2.3 Preparation of Liquid H‐PRF

For the preparation of injectable H‐PRF, venous blood (10 mL) was collected from each of the 16 participants in sterile PRF tubes without anticoagulants. After horizontal centrifugation at 700 RCF for 8 min, the upper platelet‐poor plasma (PPP) fraction (approximately 2–3 mL) from each tube was carefully collected and divided into four separate Eppendorf tubes. One aliquot served as the control (unheated), whereas the remaining three aliquots were subjected to thermal treatment at 37°C, 45°C, and 60°C for 10 min. Following heating, the albumin gel formed in the heated tubes was allowed to cool, after which it was combined with the buffy coat fraction obtained from the same donor sample to generate the injectable H‐PRF gels for each temperature condition. Thus, each donor contributed samples to all four experimental conditions, and the donor blood sample represented the primary experimental unit, whereas growth factor measurements were treated as assay outcomes for each condition.

**FIGURE 1:**
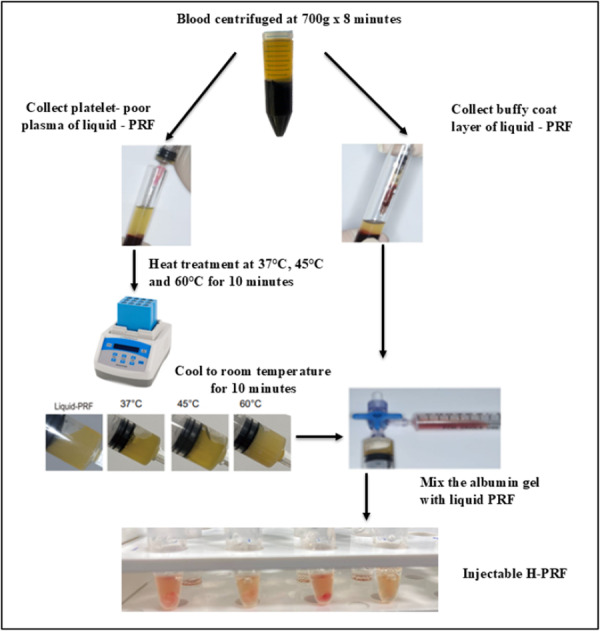
Process followed in the preparation of injectable H‐PRF.

Three of the Eppendorf tubes were heated at 37°C, 45°C, and 60°C for 10 min in an incubator. After heat treatment, the albumin gel formed was allowed to cool at room temperature for an additional 10 min to induce denaturation. The remaining tube, containing 2 mL of liquid PRF, which included the upper PRP layer and the buffy coat, was kept at room temperature and used as the control group. The buffy coat from the remaining samples was mixed with the cooled albumin gels from Groups II, III, and IV. This mixture aimed to enhance the resorption properties while increasing the concentration of cells and growth factors. A schematic workflow illustrating the preparation and allocation of samples for each temperature condition is presented in Figure 1.

### 2.4 Solidification Time

To determine the solidification time of the injectable H‐PRF gels, each group's gel was placed into separate tissue‐embedding molds immediately after preparation. The molds were tilted at a 45° angle every 15 s to monitor if the gel had dissolved. The solidification time was recorded when the gel was no longer dissolving, indicating that it had completely solidified.

### 2.5 Degradation of the H‐PRF Gel

To assess the degradation properties of the injectable H‐PRF gels, each gel sample was placed in separate tissue‐embedding molds and covered with DMEM in a humidified incubator set at 37°C with 5% CO_2_. The gels were incubated for 24 days, and the photographs and weights of the gels were recorded daily to monitor any changes in the gel's integrity or weight over time.

### 2.6 Growth Factor Analysis

The growth factor analysis was conducted using the LEGENDplex Human Growth Factor Panel V02 (BioLegend), a bead‐based multiplex assay designed to quantify multiple growth factors in a single sample. This analysis included key factors such as Angiopoietin‐2, PDGF‐AA, PDGF‐BB, TGF‐β, VEGF, EGF, and HGF. The manufacturer's instructions were followed in this assay based on fluorescence‐encoded beads coated with capture antibodies specific to the target analytes. The concentration of each analyte was quantified using a flow cytometer based on a standard curve generated from the assay.

To prepare for the analysis, 15 μL of assay buffer was added to each of the four tubes (Groups I, II, III, and IV), followed by 15 μL of premixed beads and 15 μL of detection antibodies. They were placed in an aluminum foil–covered rack to protect from light. The tubes were incubated for 2 h at room temperature on a gel rocker and shaken at 1000 rpm, after which 25 μL of SA‐PE was added to each tube. After another 30 min of incubation, the samples were again shaken at 1000 rpm and centrifuged at 1000 g for 5 min using a swinging bucket rotor with a miniplate adaptor. After discarding the supernatant, the beads were washed, resuspended, and the final samples were analyzed using a flow cytometer to differentiate the bead sizes and quantify the analytes based on the PE fluorescent signal.

### 2.7 Statistical Analysis

The data collected during the study were tabulated onto a Microsoft Excel spreadsheet. Statistical analyses were performed using Statistical Package for Social Sciences (SPSS) software, Version 21.0 for Windows (IBM Corporation, Armonk, NY). Descriptive statistics for the continuous variables were computed, with the mean and standard deviation calculated for each group. The calculation was based on a repeated‐measures one‐way ANOVA design with four conditions (control, 37°C, 45°C, and 60°C). PDGF‐AA was considered the primary outcome variable, as it represents a key growth factor associated with platelet‐mediated regenerative activity. The minimum required sample size was calculated to be 12 donors. To compensate for possible experimental variability and sample loss during laboratory procedures, 16 participants were included in the study. Differences among the four temperature conditions were evaluated using one‐way repeated‐measures ANOVA, followed by Bonferroni post‐hoc tests for pairwise comparisons.

## 3 Results

**FIGURE 2:**
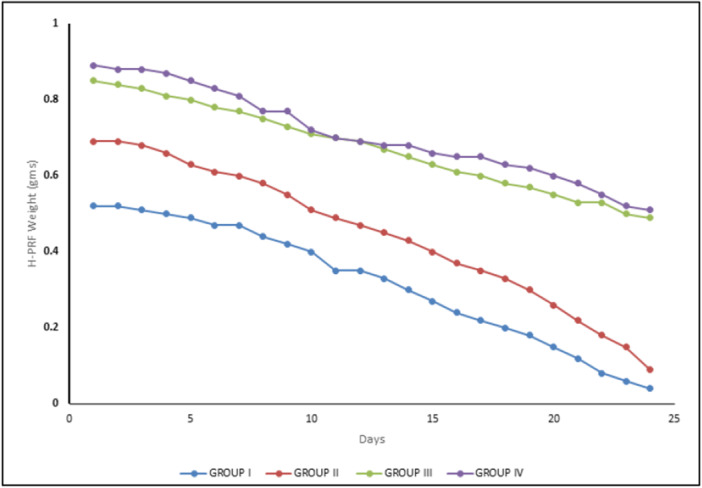
Degradation rate(s) of H‐PRF over 24 days.

The present study investigated the influence of varying heating temperatures on the solidification time, degradation profile, and release of specific growth factors in H‐PRF. The solidification time of group I was 24 min 15 s, whereas those of groups II, III, and IV were 10 min 56 s, 8 min 10 s, and 5 min, respectively. Group IV showed fastest solidification compared to all other groups. Furthermore, over the study period, the weight of the H‐PRF gels produced at different temperatures in each group decreased progressively until they were totally degraded by 24 days. The average H‐PRF weight in groups I, II, III, and IV on Day 1 was 0.52, 0.69, 0.85, and 0.89 g, respectively. After 24 days, the average H‐PRF weight of groups I, II, III, and IV reduced to 0.04, 0.09, 0.49, and 0.51 g, respectively, as shown in Figure 2.

Based on the growth factor analysis, four growth factors, Angiopoietin‐2, PDGF‐AA, Human TGF‐α, and VEGF, showed statistically significant differences in their mean concentrations among the groups studied. Angiopoietin‐2 demonstrated the most pronounced variation, with Group II showing the highest mean concentration (10.71 ± 0.83 pg/mL), followed by Group I (8.12 ± 1.75 pg/mL), Group III (7.08 ± 0.61 pg/mL), and Group IV (4.77 ± 0.11 pg/mL). This suggests that moderate heating conditions may enhance angiopoietin release. Likewise, PDGF‐AA levels varied significantly, with Group II again displaying the highest concentration (2552.16 ± 164.55 pg/mL), whereas Group IV showed the lowest concentration (1899.95 ± 68.40 pg/mL). The elevation in PDGF‐AA in Group II may imply that heating to 37°C facilitates a more optimal protein‐release environment compared to other temperature conditions. TGF‐α also showed a significant difference across groups, although the absolute mean differences were minimal, with values closely clustered around 287–289 pg/mL. Human VEGF levels were significantly different among the groups as well, with Group I (58.46 ± 1.88 pg/mL) showing the highest mean value and Group IV (55.57 ± 0.15 pg/mL) showing the lowest mean value. These findings collectively indicate that moderate heating may upregulate certain key angiogenic factors, whereas excessive heat could reduce bioactive protein release. Other growth factors, such as Human EGF, FGF‐basic, HGF, and PDGF‐BB, did not show significant differences across the groups, suggesting that their levels may be less sensitive to thermal processing conditions used in this study. EGF levels were relatively high across all groups (ranging from 3585.80 to 4834.68 pg/mL), but the variability was not statistically significant. Similarly, FGF‐basic showed consistent low‐level readings across all groups, indicating negligible thermal influence on its release (Table 1). The results of the post hoc analysis using Bonferroni post hoc test are reported in Table 2.

**TABLE 1:** Comparison of the mean levels of various human growth factors in four groups.

Growth factor	Group I (pg/mL)	Group II (pg/mL)	Group III (pg/mL)	Group IV (pg/mL)	*F*	*p* value
Angiopoietin‐2	8.12 ± 1.75	10.71 ± 0.83	7.08 ± 0.61	4.77 ± 0.11	23.580	< 0.001*
EGF	4337.08 ± 1133.13	4834.68 ± 433.68	4195.33 ± 21.92	3585.80 ± 69.97	2.863	0.081
FGF‐basic	6.60 ± 0.49	6.35 ± 0.27	6.27 ± 0.49	6.27 ± 0.31	0.589	0.634
HGF	45.32 ± 0.39	45.20 ± 0.23	45.26 ± 0.28	45.12 ± 0.17	0.356	0.786
PDGF‐AA	1950.10 ± 340.72	2552.16 ± 164.55	2092.08 ± 37.05	1899.95 ± 68.40	9.464	0.002*
PDGF‐BB	3597.31 ± 917.57	4291.50 ± 368.17	3445.39 ± 67.09	3378.29 ± 83.48	2.841	0.083
TGF‐α	288.59 ± 0.84	288.84 ± 0.96	287.41 ± 0.47	287.58 ± 0.43	3.957	0.036*
VEGF	58.46 ± 1.88	56.77 ± 0.49	56.77 ± 0.28	55.57 ± 0.15	5.841	0.011*

* *p* < 0.05 significant.

**TABLE 2:** Comparison of the mean difference in the levels of various human growth factors between the groups.

Growth factor	Groups	Mean difference	95% Confidence interval		*p* value
			Lower bound	Upper bound	
Angiopoietin‐2	Groups I vs. II	−2.585	−4.846	−0.323	0.022*
	Groups I vs. III	1.045	−1.216	3.306	1.000
	Groups I vs. IV	3.355	1.093	5.616	0.003*
	Groups II vs. III	3.630	1.368	5.891	0.002*
	Groups II vs. IV	5.940	3.678	8.201	0.000*
	Groups III vs. IV	2.310	0.048	4.571	0.044*
PDGF‐AA	Groups I vs. II	−602.062	−1032.63	−171.492	0.005*
	Groups I vs. III	−141.975	−572.545	288.595	1.000
	Groups I vs. IV	50.155	−380.415	480.725	1.000
	Groups II vs. III	460.087	29.517	890.657	0.033*
	Groups II vs. IV	652.217	221.647	1082.787	0.003*
	Groups III vs. IV	192.130	−238.440	622.700	1.000
TGF‐α	Groups I vs. II	−0.251	−1.849	1.346	1.000
	Groups I vs. III	1.174	−0.423	2.772	0.234
	Groups I vs. IV	1.006	−0.591	2.604	0.422
	Groups II vs. III	1.426	−0.172	3.024	0.094
	Groups II vs. IV	1.258	−0.339	2.856	10.173
	Groups III vs. IV	−0.167	−1.765	1.430	1.000
VEGF	Groups I vs. II	1.697	−0.497	3.892	0.188
	Groups I vs. III	1.697	−0.497	3.892	0.188
	Groups I vs. IV	2.893	0.697	5.088	0.008*
	Groups II vs. III	0	−2.195	2.195	1.000
	Groups II vs. IV	1.195	−0.999	3.391	0.670
	Groups III vs. IV	1.195	−0.999	3.391	0.670

* *p* < 0.05 significant.

## 4 Discussion

Autologous platelet concentrates such as platelet‐rich plasma (PRP) and platelet‐rich fibrin (PRF) have been widely adopted in regenerative medicine due to their ability to enhance wound healing through the release of endogenous growth factors. Their popularity is further supported by their ease of preparation, which involves simple centrifugation of the patient's own blood, making them both accessible and cost‐effective (Mohan et al. 2019). Although PRP and PRF share a common origin, they differ substantially in their preparation protocols, structural architecture, therapeutic concern, and biological activity. Notably, PRF offers several advantages over PRP: it requires no anticoagulants, allows for natural polymerization resulting in physiological thrombin levels, and forms an interconnected fibrin network that facilitates cellular migration and cytokine entrapment (Dohan Ehrenfest et al. 2009a). Furthermore, the three‐dimensional architecture of PRF contributes to its flexibility and structural integrity, forming a stable membrane.

Platelet concentrates have evolved across four generations. PRP, the first generation, was initially introduced in dental practice by Marx (Marx et al. 1998). However, the use of anticoagulants in PRP limits its biological compatibility. The second generation, PRF, developed by Choukroun, eliminated the need for anticoagulants, enhancing its clinical applicability (Choukroun et al. 2006). The third generation, comprising variants such as l‐PRF, A‐PRF, CGF, and PRGF, aimed to improve mechanical resilience, biodegradability, and the sustained release of growth factors. The fourth generation explores the integration of supportive cellular elements, such as mesenchymal stem cells, addressing limitations in cellular availability (Shirbhate and Bajaj 2022). Among these, PRF has evolved to become a cornerstone in periodontal regeneration due to its ability to accelerate healing and enhance tissue repair (Chatterjee and Debnath 2019). It is composed of a complex matrix enriched with platelets, leukocytes, and fibrin, with the matrix as a reservoir for essential growth factors, including VEGF, EGF, PDGF‐AA, FGF, PDGF‐BB, and TGF‐α, contributing significantly to hard and soft tissue repair (Chatterjee and Debnath 2019; Masuki et al. 2016). The biological basis for PRF's efficacy lies in the α‐granules of platelets, which serve as a pool for a variety of growth factors that are chemotactic and mitogenic, thereby regulating cell proliferation, migration, and tissue regeneration (Dohan Ehrenfest et al. 2009b). Importantly, PRF's properties are highly dependent on centrifugation parameters such as rotor angulation, radius size, relative centrifugal force (RCF), and tube composition.

One of the variants, i‐PRF, developed through low‐speed centrifugation, retains a liquid consistency for a short duration before coagulating. This enables it to be mixed with biomaterials like bone grafts and collagen membranes, forming a cohesive matrix within seconds (Amin et al. 2022). However, a standard angulated or fixed‐angle centrifugation system imposes several limitations: the cell distribution is uneven, smaller cells may not reach upper layers efficiently, and cells experience shear stress due to higher g‐forces, potentially affecting viability. To address these issues, horizontal centrifugation has been introduced. This method utilizes swing‐out bucket rotors for more precise density‐based separation of blood components (Miron et al. 2019; Wu et al. 2023). The resultant horizontal PRF (H‐PRF) shows improved growth factor concentration and enhanced regenerative outcomes due to better structural homogeneity.

Injectable horizontal platelet‐rich fibrin (H‐PRF) represents an advanced form of PRF, characterized by its favorable cellular viability and fibrin architecture. Although increasing the heating temperature leads to a proportional increase in the dead cell count, more than 90% of cells in H‐PRF gels remain viable across all temperature groups (Zheng et al. 2022). Furthermore, liquid H‐PRF has a loosely organized fibrin network that efficiently entraps platelets and leukocytes, contributing to its enhanced regenerative potential (Feng et al. 2022). Horizontal centrifugation significantly enhances the regenerative profile of PRF by improving both cellular yield and growth factor release. Compared to fixed‐angle PRF variants (l‐PRF, A‐PRF, Alb‐PRF), H‐PRF achieves up to a fourfold increase in the total cell concentration—particularly leukocytes—and consistently yields higher levels of PDGF‐BB, VEGF, and FGF‐2. These growth factors are released more uniformly and sustainably across the fibrin matrix, whereas pro‐inflammatory cytokines such as IL‐6 and IL‐1β are notably reduced. The even cellular distribution and preserved integrity of platelets and leukocytes in H‐PRF contribute to superior bioactivity, immunomodulation, and regenerative efficacy, supporting its preferential use in clinical protocol (Farshidfar et al. 2025).

Despite these advances, i‐PRF still presents challenges, particularly its rapid degradation and limited mechanical stability. Although modifications to centrifugation protocols and combination with other biomaterials have been attempted, biosafety concerns remain unresolved. Recent research has focused on thermal conditioning of the plasma fraction post‐centrifugation to improve PRF's stability. Heating the plasma at various temperatures for 10 min, followed by mixing with the buffy coat‐rich liquid PRF, produces a robust, growth factor‐rich H‐PRF gel suitable for injectable applications (Shirakata et al. 2021; Zheng et al. 2022; Fujioka‐Kobayashi et al. 2020).

However, to date, literature exploring the precise impact of thermal treatment on growth factor profiles and mechanical characteristics of injectable H‐PRF remains scarce. The present study sought examine the effect of heating injectable H‐PRF at 37°C, 45°C, and 60°C, alongside a control group. These temperatures were selected based on existing literature suggesting that fibrinogen denatures between 45°C and 60°C, altering matrix properties (Wu et al. 2023). Zheng et al. demonstrated that heating increases PRF gel density and improves mechanical properties, with over 90% cell viability retained at certain temperature thresholds (Zheng et al. 2022). Similarly, Wu et al. reported that increasing the temperature beyond 50°C makes the fibrin network dense, but reduces cellular viability and elasticity (Wu et al. 2023). In the present study, the solidification time and degradation profiles were found to be significantly affected by temperature. H‐PRF heated at 60°C showed the fastest solidification (5 min), whereas non‐heated H‐PRF showed the longest solidification (24 min 15 s). These findings align with previous observations where PRF heated at 60°C solidified in 5.03 min versus 44.5 min for unheated PRF (Zheng et al. 2022). Our findings also echo Wu et al.'s conclusion that fibrin becomes denser and more stable at higher temperatures (Wu et al. 2023).

Degradation assessments over 24 days revealed that H‐PRF heated at 60°C had the highest initial mass (0.89 g), followed by H‐PRF heated at 45°C (0.76 g), suggesting denser matrix formation at elevated temperatures. By Day 24, non‐heated H‐PRF and H‐PRF heated at 37°C showed almost complete degradation, whereas H‐PRF heated at 60°C and 45°C retained 0.49 and 0.51 g, respectively. These trends reflect findings from previous studies supporting the role of thermal treatment in delaying biodegradation (Zheng et al. 2022; Kawase et al. 2014).

Growth factor analysis using the LEGENDplex Human Growth Factor Panel showed statistically significant intergroup differences in Angiopoietin‐2, PDGF‐AA, TGF‐α, and VEGF (*p* < 0.05). Notably, VEGF levels differed significantly between Groups I and IV, highlighting temperature‐related effects on angiogenic potential. Other growth factors such as EGF, FGF, HGF, and PDGF‐BB showed no significant differences across groups. These observations suggest that mild to moderate heating enhances the release of some growth factors, whereas excessive heat may compromise protein activity. The increased levels of Angiopoietin‐2, PDGF‐AA, and VEGF in moderately heated groups point to a favorable balance between improved mechanical strength and biological functionality. Our findings are consistent with Kobayashi et al. reporting a sustained release of PDGF, VEGF, and IGF‐1 from albumin‐based PRF over several days (Kobayashi et al. 2016). Additionally, Kargarpour et al emphasized the importance of mixing heated PPP with cell‐rich PRF to maintain growth factor activity (Kargarpour et al. 2020). Overall, this study shows that the regenerative potential of the H‐PRF can be modulated using heat treatment.

There are some limitations within our in vitro study. Although our growth factor analysis was limited to a single time point, future studies could involve serial sampling to capture dynamic changes in growth factor kinetics. Additionally, inclusion of participants of various age groups could aid in the evaluation of the variations in growth factors' release based on age. Nonetheless, the findings from this study affirm that thermally modified H‐PRF can significantly influence both mechanical and biological properties.

## 5 Conclusion

Based on our findings, it can be concluded that thermal treatment of injectable H‐PRF at 45°C and 60°C enhances the mechanical properties, whereas H‐PRF treated at 37°C and the non‐heated control better preserve growth factor levels. Consequently, customized heat‐treated PRF formulations can be utilized based on specific clinical applications. Continued investigation into temperature‐dependent modifications will be critical for maximizing the regenerative potential of platelet‐enriched biomaterials.

## Author Contributions

Conceptualization: Deshetti Ketan, Anita Kulloli, Sharath Shetty, Santosh Martande, Avinash Sanap. Methodology: Deshetti Ketan, Shivani Yerte, Avinash Sanap. Formal analysis: Deshetti Ketan, Anita Kulloli, Sharath Shetty, Santosh Martande, Dileep Sharma. Investigation: Deshetti Ketan, Shivani Yerte, Avinash Sanap, Shubham Lawate, Chaitaly Munot. Resources: Anita Kulloli, Sharath Shetty, Santosh Martande, Avinash Sanap, Dileep Sharma. Data curation: Deshetti Ketan, Anita Kulloli. Writing – original draft preparation: Deshetti Ketan, Anita Kulloli. Writing – review and editing: Deshetti Ketan, Anita Kulloli, Sharath Shetty, Santosh Martande, Dileep Sharma. Supervision: Anita Kulloli, Sharath Shetty, Santosh Martande. All authors have read and agreed to the published version of the manuscript.

## Funding

The authors have nothing to report.

## Ethics Statement

This study was conducted following the ethical standards outlined in the Helsinki Declaration on Medical Protocol and Ethics. The study protocol was approved by the Dr. D.Y. Patil Vidyapeeth Ethics Committee (Approval number‐DPU/806/2/2023).

## Consent

Informed consent was obtained from all subjects involved in the study.

## Conflicts of Interest

The authors declare no conflicts of interest.

## Data Availability

The data that support the findings of this study are available from the corresponding author upon reasonable request.
